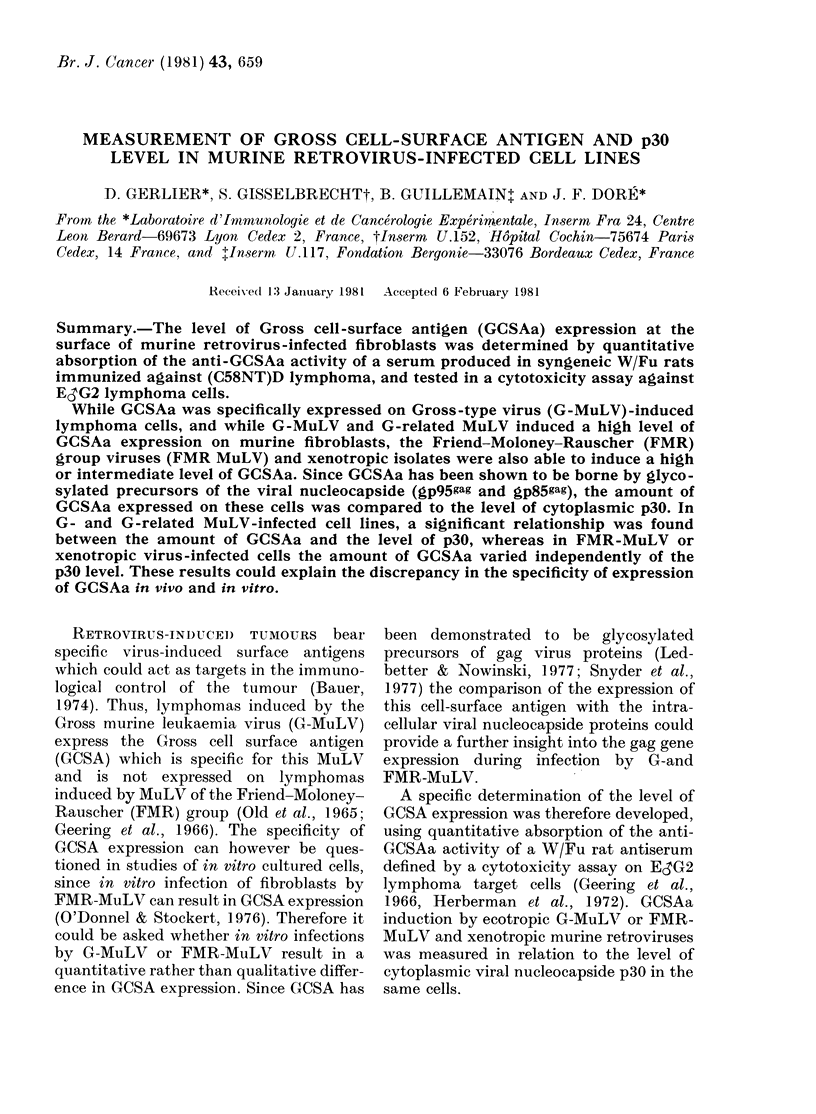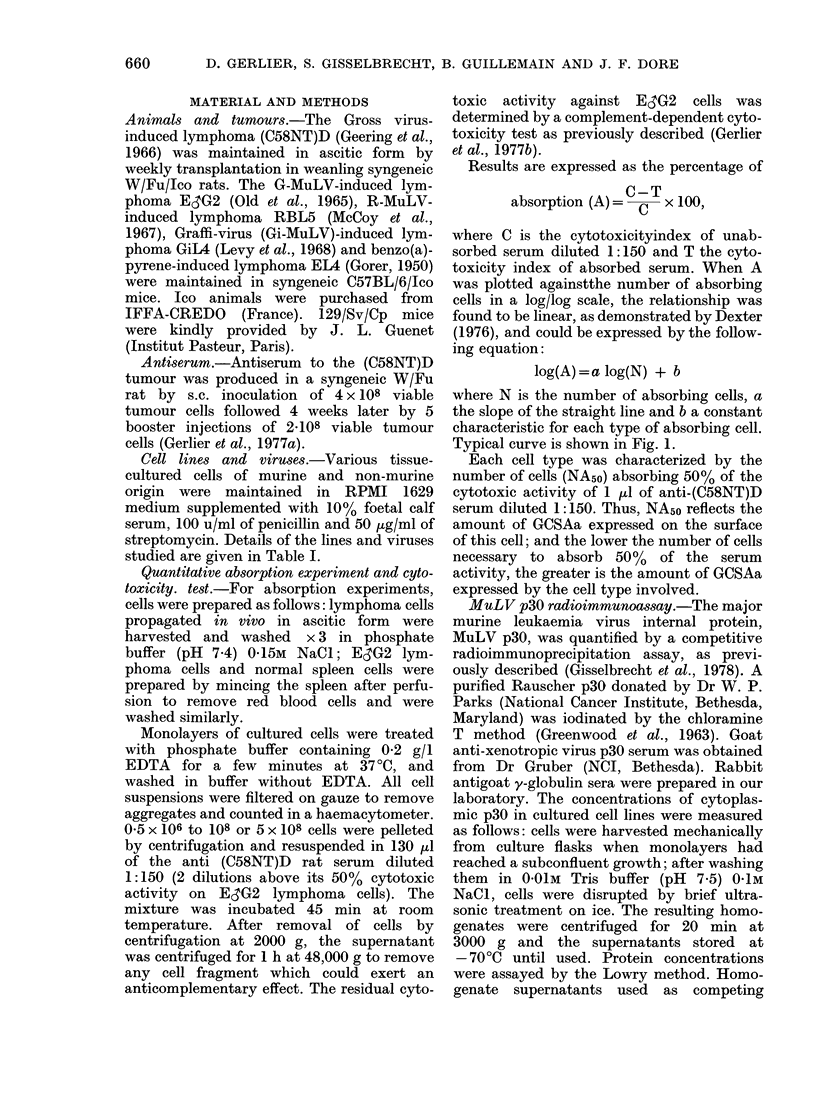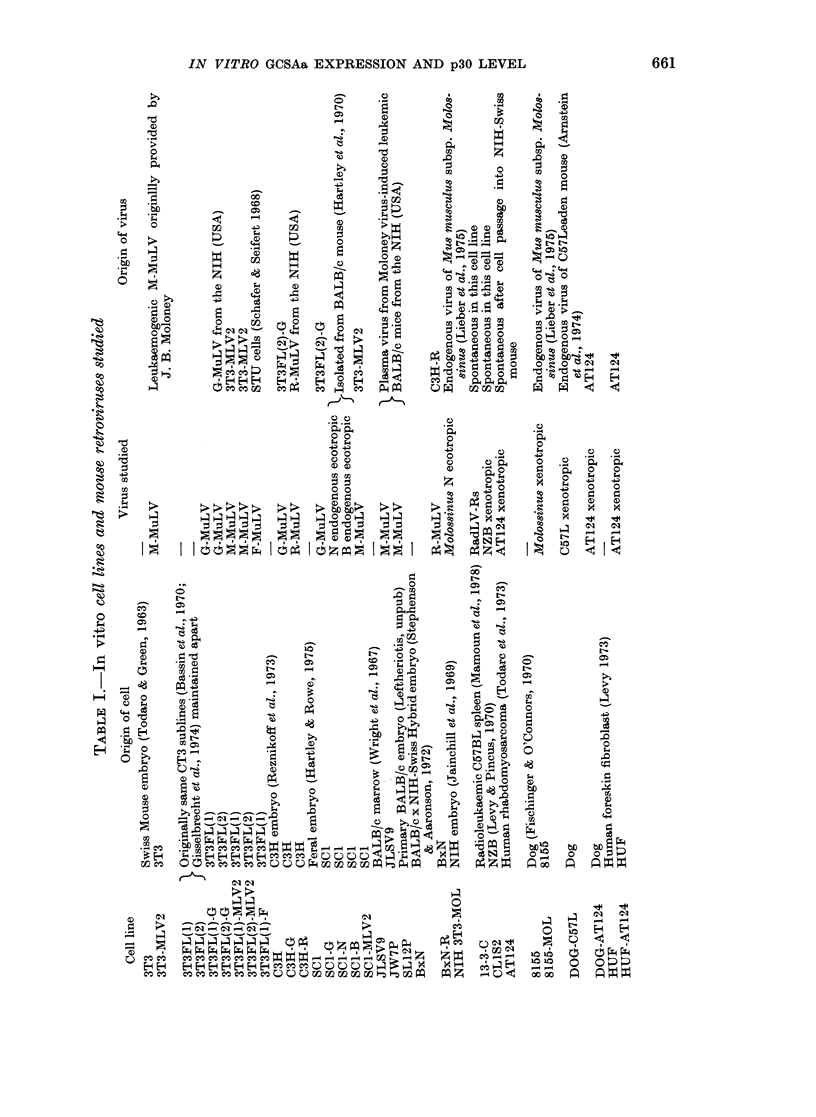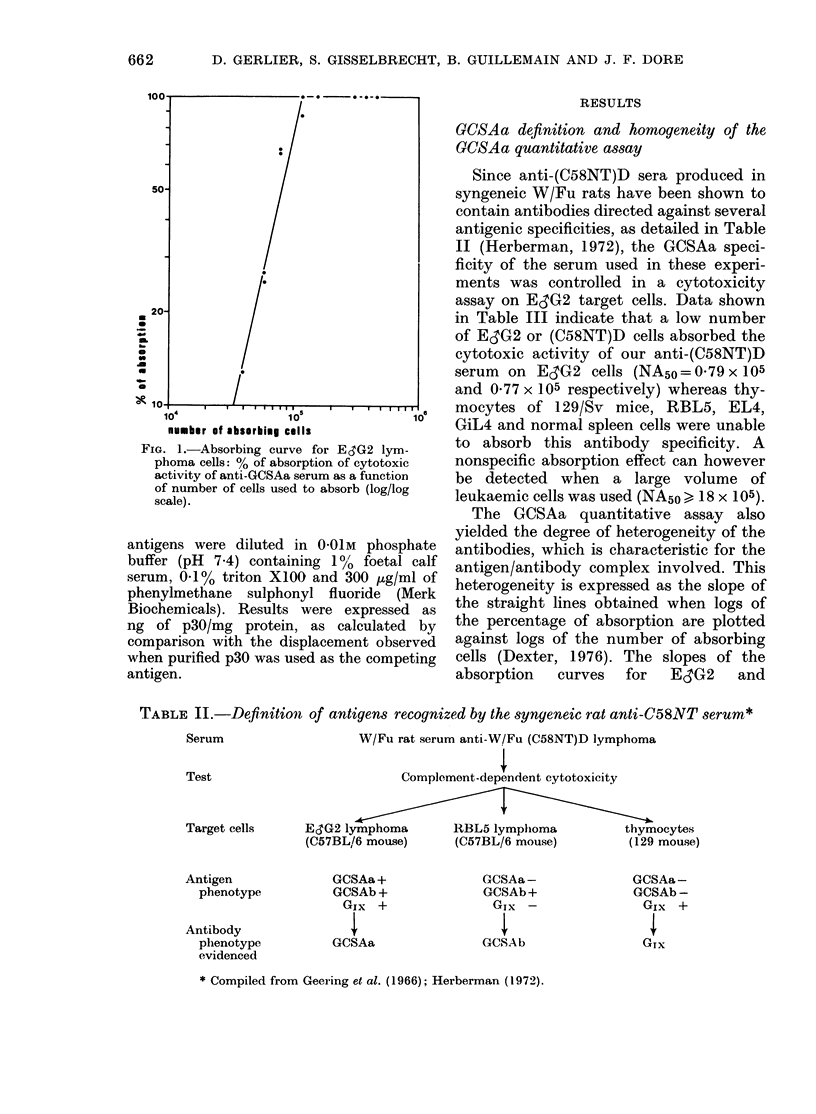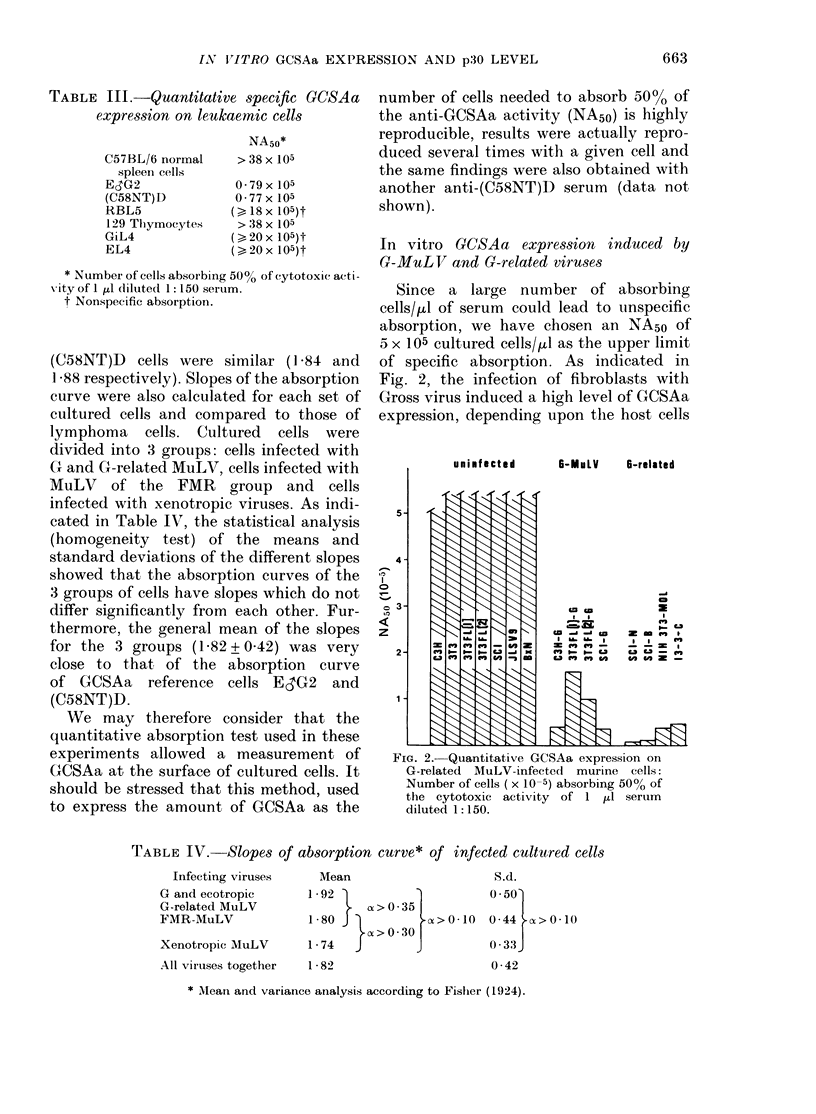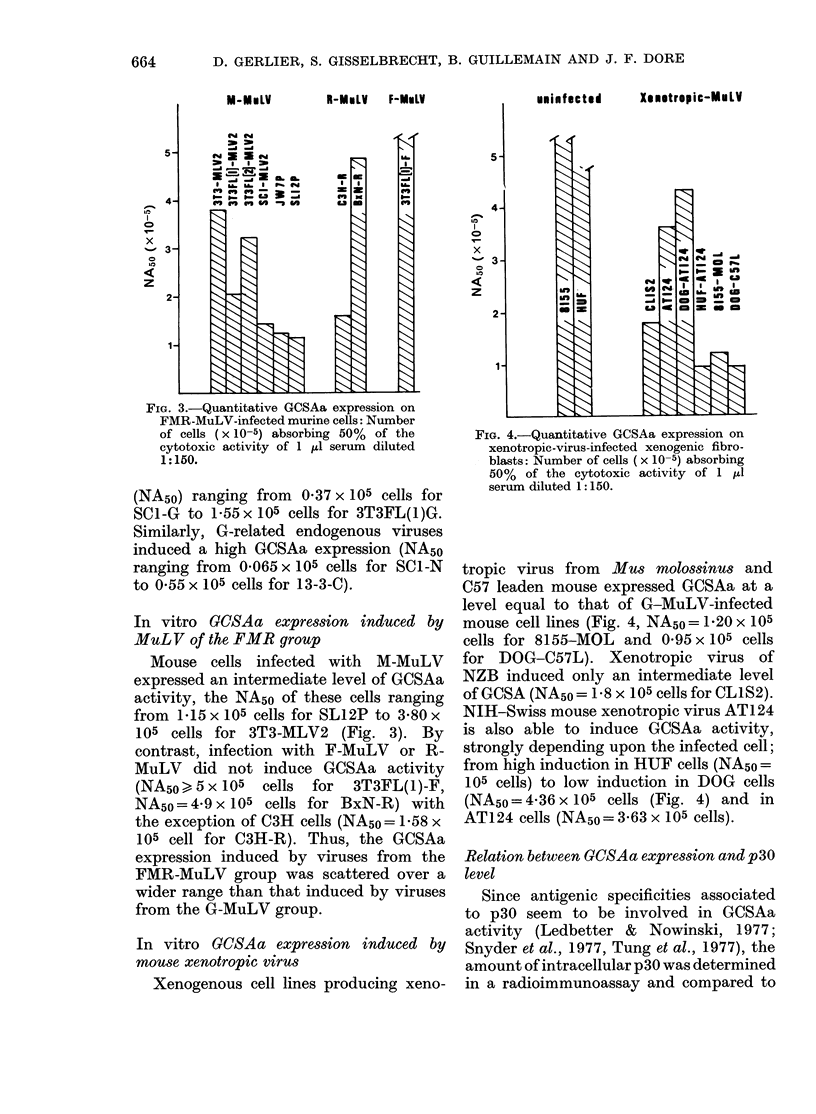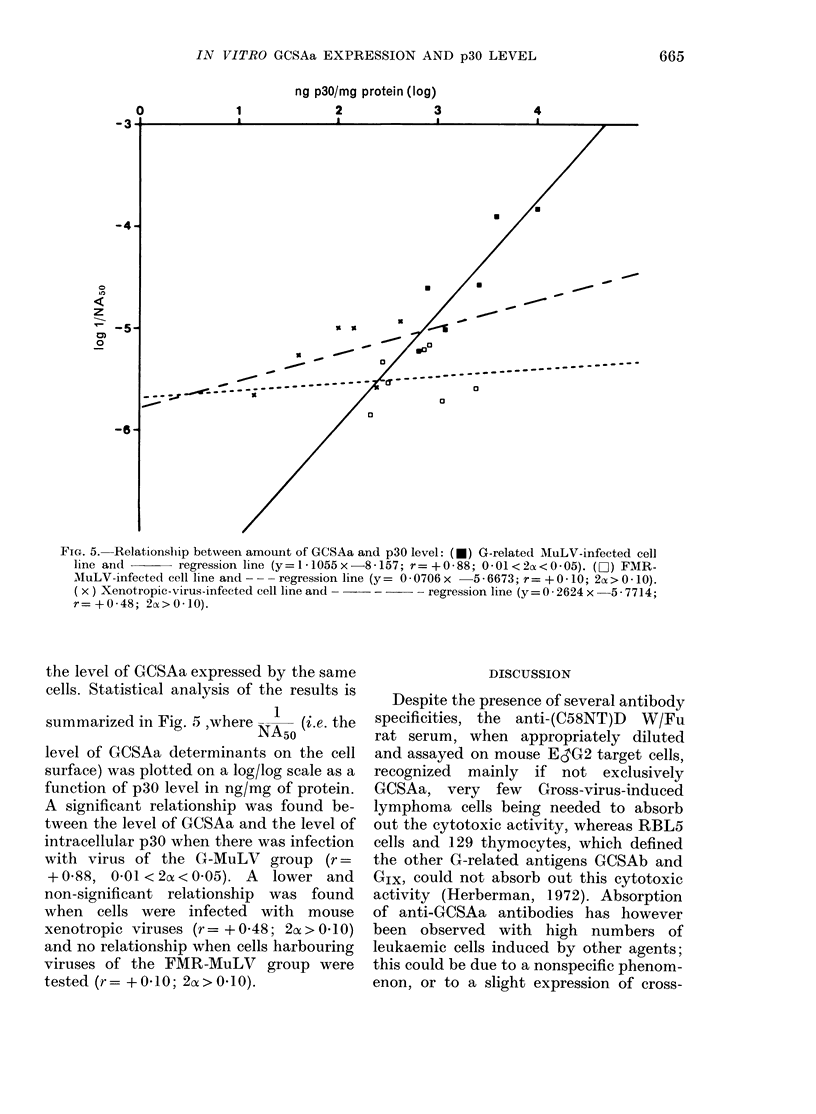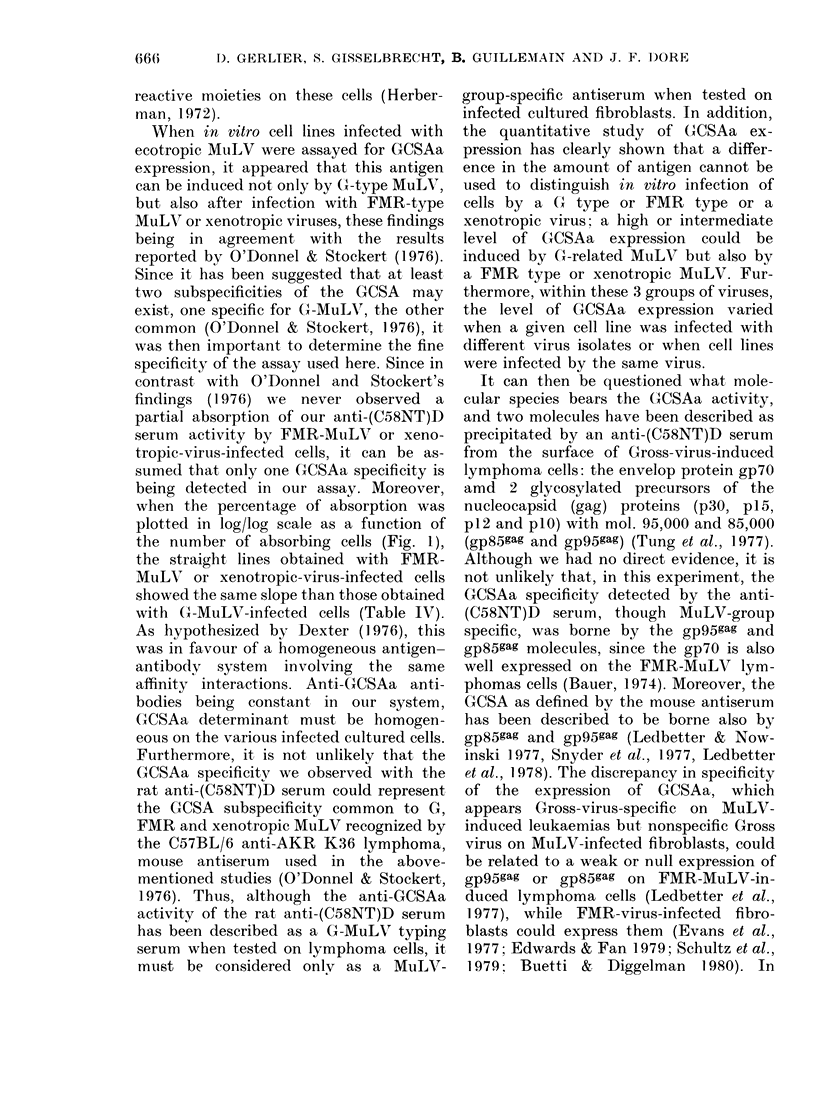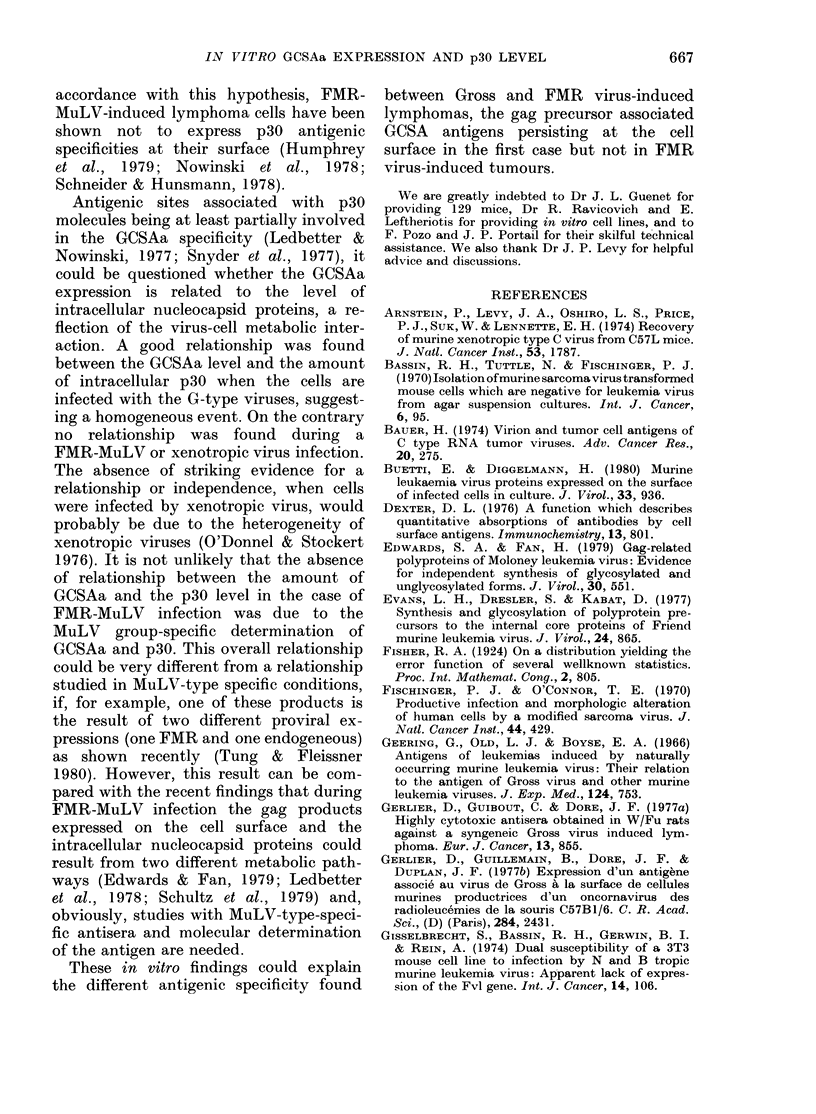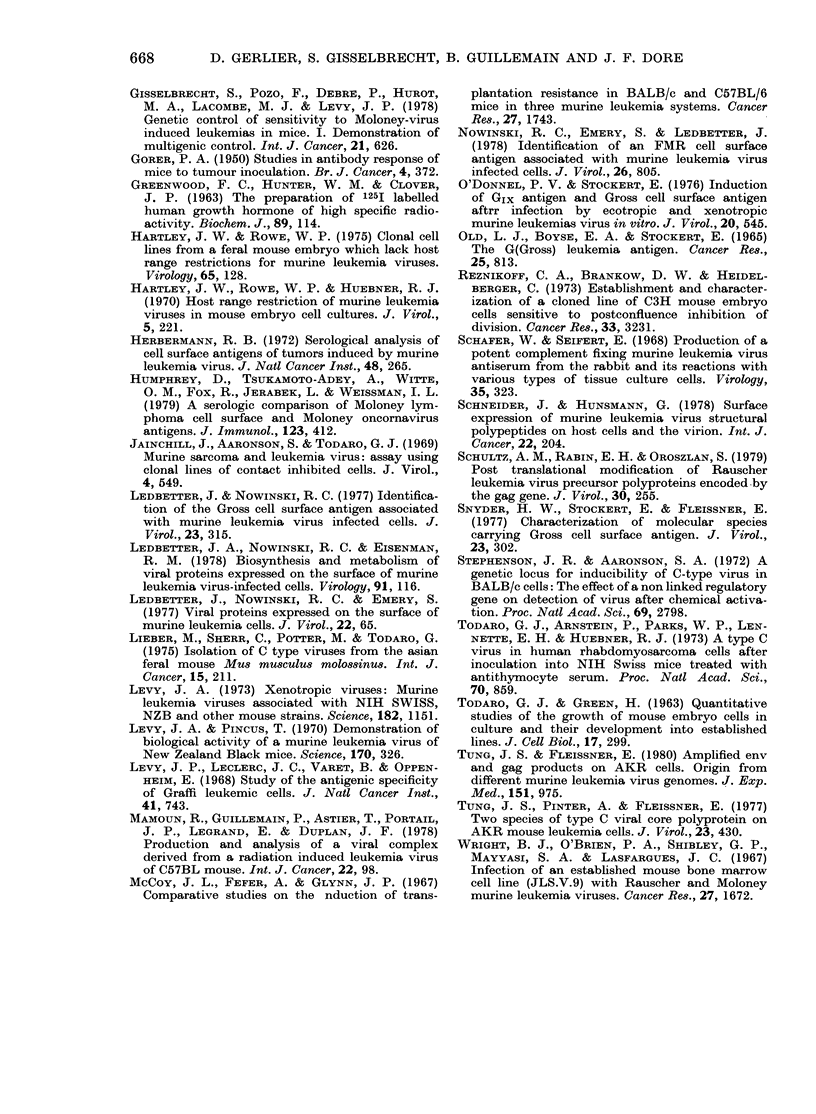# Measurement of Gross cell-surface antigen and p30 level in murine retrovirus-infected cell lines.

**DOI:** 10.1038/bjc.1981.97

**Published:** 1981-05

**Authors:** D. Gerlier, S. Gisselbrecht, B. Guillemain, J. F. Doré

## Abstract

The level of Gross cell-surface antigen (GCSAa) expression at the surface of murine retrovirus-infected fibroblasts was determined by quantitative absorption of the anti-GCSAa activity of a serum produced in syngeneic W/Fu rats immunized against (C58NT)D lymphoma, and tested in a cytotoxicity assay against E male G2 lymphoma cells. While GCSAa was specifically expressed on Gross-type virus (G-MuLV)-induced lymphoma cells, and while G-MuLV and G-related MuLV induced a high level of GCSAa expression on murine fibroblasts, the Friend-Moloney-Rauscher (FMR) group viruses (FMR MuLV) and xenotropic isolates were also able to induce a high or intermediate level of GCSAa. Since GCSAa has been shown to be borne by glycosylated precursors of the viral nucleocapside (gp95gag and gp85gag), the amount of GCSAa expressed on these cells was compared to the level of cytoplasmic p30. In G- and G-related MuLV-infected cell lines, a significant relationship was found between the amount of GCSAa and the level of p30, whereas in FMR-MuLV or xenotropic virus-infected cells the amount of GCSAa varied independently of the p30 level. These results could explain the discrepancy in the specificity of expression of GCSAa in vivo and in vitro.


*Br. J. Cancer* (1981) **43**, 659

### MEASUREMENT OF GROSS CELL-SURFACE ANTIGEN AND p30 LEVEL IN MURINE RETROVIRUS-INFECTED CELL LINES

D. GERLIER*, S. GISSELBRECHT†, B. GUILLEMAIN‡ AND J. F. DORÉ*

*From the *Laboratoire d'Immunologie et de Cancérologie Expérimentale, Inserm Fra 24, Centre Leon Berard—69673 Lyon Cedex 2, France, †Inserm U.152, Hôpital Cochin—75674 Paris Cedex, 14 France, and ‡Inserm U.117, Fondation Bergonie—33076 Bordeaux Cedex, France*

Received 13 January 1981 Accepted 6 February 1981

**Summary.—The level of Gross cell-surface antigen (GCSAa) expression at the surface of murine retrovirus-infected fibroblasts was determined by quantitative absorption of the anti-GCSAa activity of a serum produced in syngeneic W/Fu rats immunized against (C58NT)D lymphoma, and tested in a cytotoxicity assay against E♂G2 lymphoma cells.**

**While GCSAa was specifically expressed on Gross-type virus (G-MuLV)-induced lymphoma cells, and while G-MuLV and G-related MuLV induced a high level of GCSAa expression on murine fibroblasts, the Friend–Moloney–Rauscher (FMR) group viruses (FMR MuLV) and xenotropic isolates were also able to induce a high or intermediate level of GCSAa. Since GCSAa has been shown to be borne by glycosylated precursors of the viral nucleocapside ($gp95^{gag}$ and $gp85^{gag}$), the amount of GCSAa expressed on these cells was compared to the level of cytoplasmic p30. In G- and G-related MuLV-infected cell lines, a significant relationship was found between the amount of GCSAa and the level of p30, whereas in FMR-MuLV or xenotropic virus-infected cells the amount of GCSAa varied independently of the p30 level. These results could explain the discrepancy in the specificity of expression of GCSAa *in vivo* and *in vitro*.**

RETROVIRUS-INDUCED TUMOURS bear specific virus-induced surface antigens which could act as targets in the immunological control of the tumour (Bauer, 1974). Thus, lymphomas induced by the Gross murine leukaemia virus (G-MuLV) express the Gross cell surface antigen (GCSA) which is specific for this MuLV and is not expressed on lymphomas induced by MuLV of the Friend–Moloney–Rauscher (FMR) group (Old *et al.*, 1965; Geering *et al.*, 1966). The specificity of GCSA expression can however be questioned in studies of *in vitro* cultured cells, since *in vitro* infection of fibroblasts by FMR-MuLV can result in GCSA expression (O'Donnel & Stockert, 1976). Therefore it could be asked whether *in vitro* infections by G-MuLV or FMR-MuLV result in a quantitative rather than qualitative difference in GCSA expression. Since GCSA has been demonstrated to be glycosylated precursors of gag virus proteins (Ledbetter & Nowinski, 1977; Snyder *et al.*, 1977) the comparison of the expression of this cell-surface antigen with the intracellular viral nucleocapside proteins could provide a further insight into the gag gene expression during infection by G-and FMR-MuLV.

A specific determination of the level of GCSA expression was therefore developed, using quantitative absorption of the anti-GCSAa activity of a W/Fu rat antiserum defined by a cytotoxicity assay on E♂G2 lymphoma target cells (Geering *et al.*, 1966, Herberman *et al.*, 1972). GCSAa induction by ecotropic G-MuLV or FMR-MuLV and xenotropic murine retroviruses was measured in relation to the level of cytoplasmic viral nucleocapside p30 in the same cells.

### MATERIAL AND METHODS

*Animals and tumours.*—The Gross virus-induced lymphoma (C58NT)D (Geering *et al.*, 1966) was maintained in ascitic form by weekly transplantation in weanling syngeneic W/Fu/Ico rats. The G-MuLV-induced lymphoma E♂G2 (Old *et al.*, 1965), R-MuLV-induced lymphoma RBL5 (McCoy *et al.*, 1967), Graffi-virus (Gi-MuLV)-induced lymphoma GiL4 (Levy *et al.*, 1968) and benzo(a)-pyrene-induced lymphoma EL4 (Gorer, 1950) were maintained in syngeneic C57BL/6/Ico mice. Ico animals were purchased from IFFA-CREDO (France). 129/Sv/Cp mice were kindly provided by J. L. Guenet (Institut Pasteur, Paris).

*Antiserum.*—Antiserum to the (C58NT)D tumour was produced in a syngeneic W/Fu rat by s.c. inoculation of $4 \times 10^8$ viable tumour cells followed 4 weeks later by 5 booster injections of $2 \cdot 10^8$ viable tumour cells (Gerlier *et al.*, 1977*a*).

*Cell lines and viruses.*—Various tissue-cultured cells of murine and non-murine origin were maintained in RPMI 1629 medium supplemented with 10% foetal calf serum, 100 u/ml of penicillin and 50 μg/ml of streptomycin. Details of the lines and viruses studied are given in Table I.

*Quantitative absorption experiment and cytotoxicity. test.*—For absorption experiments, cells were prepared as follows: lymphoma cells propagated *in vivo* in ascitic form were harvested and washed ×3 in phosphate buffer (pH 7·4) 0·15M NaCl; E♂G2 lymphoma cells and normal spleen cells were prepared by mincing the spleen after perfusion to remove red blood cells and were washed similarly.

Monolayers of cultured cells were treated with phosphate buffer containing 0·2 g/l EDTA for a few minutes at 37°C, and washed in buffer without EDTA. All cell suspensions were filtered on gauze to remove aggregates and counted in a haemacytometer. $0{\cdot}5 \times 10^6$ to $10^8$ or $5 \times 10^8$ cells were pelleted by centrifugation and resuspended in 130 μl of the anti (C58NT)D rat serum diluted 1:150 (2 dilutions above its 50% cytotoxic activity on E♂G2 lymphoma cells). The mixture was incubated 45 min at room temperature. After removal of cells by centrifugation at 2000 g, the supernatant was centrifuged for 1 h at 48,000 g to remove any cell fragment which could exert an anticomplementary effect. The residual cytotoxic activity against E♂G2 cells was determined by a complement-dependent cytotoxicity test as previously described (Gerlier *et al.*, 1977*b*).

Results are expressed as the percentage of

$$\text{absorption (A)} = \frac{C-T}{C} \times 100,$$

where C is the cytotoxicityindex of unabsorbed serum diluted 1:150 and T the cytotoxicity index of absorbed serum. When A was plotted againstthe number of absorbing cells in a log/log scale, the relationship was found to be linear, as demonstrated by Dexter (1976), and could be expressed by the following equation:

$$\log(A) = a \log(N) + b$$

where N is the number of absorbing cells, $a$ the slope of the straight line and $b$ a constant characteristic for each type of absorbing cell. Typical curve is shown in Fig. 1.

Each cell type was characterized by the number of cells ($NA_{50}$) absorbing 50% of the cytotoxic activity of 1 μl of anti-(C58NT)D serum diluted 1:150. Thus, $NA_{50}$ reflects the amount of GCSAa expressed on the surface of this cell; and the lower the number of cells necessary to absorb 50% of the serum activity, the greater is the amount of GCSAa expressed by the cell type involved.

*MuLV p30 radioimmunoassay.*—The major murine leukaemia virus internal protein, MuLV p30, was quantified by a competitive radioimmunoprecipitation assay, as previously described (Gisselbrecht *et al.*, 1978). A purified Rauscher p30 donated by Dr W. P. Parks (National Cancer Institute, Bethesda, Maryland) was iodinated by the chloramine T method (Greenwood *et al.*, 1963). Goat anti-xenotropic virus p30 serum was obtained from Dr Gruber (NCI, Bethesda). Rabbit antigoat γ-globulin sera were prepared in our laboratory. The concentrations of cytoplasmic p30 in cultured cell lines were measured as follows: cells were harvested mechanically from culture flasks when monolayers had reached a subconfluent growth; after washing them in 0·01M Tris buffer (pH 7·5) 0·1M NaCl, cells were disrupted by brief ultrasonic treatment on ice. The resulting homogenates were centrifuged for 20 min at 3000 g and the supernatants stored at −70°C until used. Protein concentrations were assayed by the Lowry method. Homogenate supernatants used as competing

TABLE I.—In vitro *cell lines and mouse retroviruses studied*

| Cell line | Origin of cell | Virus studied | Origin of virus |
|---|---|---|---|
| 3T3 | Swiss Mouse embryo (Todaro & Green, 1963) | — | |
| 3T3-MLV2 | 3T3 | M-MuLV | Leukaemogenic M-MuLV originllly provided by J. B. Moloney |
| 3T3FL(1) | Originally same CT3 sublines (Bassin *et al.*, 1970; Gisselbrecht *et al.*, 1974) maintained apart | — | |
| 3T3FL(2) | | — | |
| 3T3FL(1)-G | 3T3FL(1) | G-MuLV | |
| 3T3FL(2)-G | 3T3FL(2) | G-MuLV | G-MuLV from the NIH (USA) |
| 3T3FL(1)-MLV2 | 3T3FL(1) | M-MuLV | 3T3-MLV2 |
| 3T3FL(2)-MLV2 | 3T3FL(2) | M-MuLV | 3T3-MLV2 |
| 3T3FL(1)-F | 3T3FL(1) | F-MuLV | STU cells (Schafer & Seifert 1968) |
| C3H | C3H embryo (Reznikoff *et al.*, 1973) | — | |
| C3H-G | C3H | G-MuLV | 3T3FL(2)-G |
| C3H-R | C3H | R-MuLV | R-MuLV from the NIH (USA) |
| SC1 | Feral embryo (Hartley & Rowe, 1975) | — | |
| SC1-G | SC1 | G-MuLV | 3T3FL(2)-G |
| SC1-N | SC1 | N endogenous ecotropic | Isolated from BALB/c mouse (Hartley *et al.*, 1970) |
| SC1-B | SC1 | B endogenous ecotropic | |
| SC1-MLV2 | SC1 | M-MuLV | 3T3-MLV2 |
| JLSV9 | BALB/c marrow (Wright *et al.*, 1967) | — | |
| JW7P | JLSV9 | M-MuLV | Plasma virus from Moloney virus-induced leukemic BALB/c mice from the NIH (USA) |
| SL12P | Primary BALB/c embryo (Leftheriotis, unpub) | M-MuLV | |
| BxN | BALB/c x NIH-Swiss Hybrid embryo (Stephenson & Aaronson, 1972) | — | |
| BxN-R | BxN | R-MuLV | C3H-R |
| NIH 3T3-MOL | NIH embryo (Jainchill *et al.*, 1969) | *Molossinus* N ecotropic | Endogenous virus of *Mus musculus* subsp. *Molossinus* (Lieber *et al.*, 1975) |
| 13-3-C | Radioleukaemic C57BL spleen (Mamoun *et al.*, 1978) | RadLV-Rs | Spontaneous in this cell line |
| CL1S2 | NZB (Levy & Pincus, 1970) | NZB xenotropic | Spontaneous in this cell line |
| AT124 | Human rhabdomyosarcoma (Todarc *et al.*, 1973) | AT124 xenotropic | Spontaneous after cell passage into NIH-Swiss mouse |
| 8155 | Dog (Fischinger & O'Connors, 1970) | — | |
| 8155-MOL | 8155 | *Molossinus* xenotropic | Endogenous virus of *Mus musculus* subsp. *Molossinus* (Lieber *et al.*, 1975) |
| DOG-C57L | Dog | C57L xenotropic | Endogenous virus of C57Leaden mouse (Arnstein *et al.*, 1974) |
| DOG-AT124 | Dog | AT124 xenotropic | AT124 |
| HUF | Human foreskin fibroblast (Levy 1973) | — | |
| HUF-AT124 | HUF | AT124 xenotropic | AT124 |

FIG. 1.—Absorbing curve for E♂G2 lymphoma cells: % of absorption of cytotoxic activity of anti-GCSAa serum as a function of number of cells used to absorb (log/log scale).

antigens were diluted in 0·01M phosphate buffer (pH 7·4) containing 1% foetal calf serum, 0·1% triton X100 and 300 μg/ml of phenylmethane sulphonyl fluoride (Merk Biochemicals). Results were expressed as ng of p30/mg protein, as calculated by comparison with the displacement observed when purified p30 was used as the competing antigen.

### RESULTS

### *GCSAa definition and homogeneity of the GCSAa quantitative assay*

Since anti-(C58NT)D sera produced in syngeneic W/Fu rats have been shown to contain antibodies directed against several antigenic specificities, as detailed in Table II (Herberman, 1972), the GCSAa specificity of the serum used in these experiments was controlled in a cytotoxicity assay on E♂G2 target cells. Data shown in Table III indicate that a low number of E♂G2 or (C58NT)D cells absorbed the cytotoxic activity of our anti-(C58NT)D serum on E♂G2 cells ($NA_{50} = 0{\cdot}79 \times 10^5$ and $0{\cdot}77 \times 10^5$ respectively) whereas thymocytes of 129/Sv mice, RBL5, EL4, GiL4 and normal spleen cells were unable to absorb this antibody specificity. A nonspecific absorption effect can however be detected when a large volume of leukaemic cells was used ($NA_{50} \geqslant 18 \times 10^5$).

The GCSAa quantitative assay also yielded the degree of heterogeneity of the antibodies, which is characteristic for the antigen/antibody complex involved. This heterogeneity is expressed as the slope of the straight lines obtained when logs of the percentage of absorption are plotted against logs of the number of absorbing cells (Dexter, 1976). The slopes of the absorption curves for E♂G2 and

TABLE II.—*Definition of antigens recognized by the syngeneic rat anti-C58NT serum**

| | | | |
|---|---|---|---|
| Serum | W/Fu rat serum anti-W/Fu (C58NT)D lymphoma | | |
| | ↓ | | |
| Test | Complement-dependent cytotoxicity | | |
| | ↙ | ↓ | ↘ |
| Target cells | E♂G2 lymphoma (C57BL/6 mouse) | RBL5 lymphoma (C57BL/6 mouse) | thymocytes (129 mouse) |
| Antigen phenotype | GCSAa+ GCSAb+ $G_{IX}$ + | GCSAa− GCSAb+ $G_{IX}$ − | GCSAa− GCSAb− $G_{IX}$ + |
| | ↓ | ↓ | ↓ |
| Antibody phenotype evidenced | GCSAa | GCSAb | $G_{IX}$ |

* Compiled from Geering *et al.* (1966); Herberman (1972).

TABLE III.—*Quantitative specific GCSAa expression on leukaemic cells*

| | $NA_{50}$* |
|---|---|
| C57BL/6 normal spleen cells | $>38\times10^5$ |
| E♂G2 | $0\cdot79\times10^5$ |
| (C58NT)D | $0\cdot77\times10^5$ |
| RBL5 | $(\geqslant18\times10^5)$† |
| 129 Thymocytes | $>38\times10^5$ |
| GiL4 | $(\geqslant20\times10^5)$† |
| EL4 | $(\geqslant20\times10^5)$† |

* Number of cells absorbing 50% of cytotoxic activity of 1 μl diluted 1:150 serum.

† Nonspecific absorption.

(C58NT)D cells were similar (1·84 and 1·88 respectively). Slopes of the absorption curve were also calculated for each set of cultured cells and compared to those of lymphoma cells. Cultured cells were divided into 3 groups: cells infected with G and G-related MuLV, cells infected with MuLV of the FMR group and cells infected with xenotropic viruses. As indicated in Table IV, the statistical analysis (homogeneity test) of the means and standard deviations of the different slopes showed that the absorption curves of the 3 groups of cells have slopes which do not differ significantly from each other. Furthermore, the general mean of the slopes for the 3 groups ($1\cdot82\pm0\cdot42$) was very close to that of the absorption curve of GCSAa reference cells E♂G2 and (C58NT)D.

We may therefore consider that the quantitative absorption test used in these experiments allowed a measurement of GCSAa at the surface of cultured cells. It should be stressed that this method, used to express the amount of GCSAa as the number of cells needed to absorb 50% of the anti-GCSAa activity ($NA_{50}$) is highly reproducible, results were actually reproduced several times with a given cell and the same findings were also obtained with another anti-(C58NT)D serum (data not shown).

### In vitro *GCSAa expression induced by G-MuLV and G-related viruses*

Since a large number of absorbing cells/μl of serum could lead to unspecific absorption, we have chosen an $NA_{50}$ of $5\times10^5$ cultured cells/μl as the upper limit of specific absorption. As indicated in Fig. 2, the infection of fibroblasts with Gross virus induced a high level of GCSAa expression, depending upon the host cells

FIG. 2.—Quantitative GCSAa expression on G-related MuLV-infected murine cells: Number of cells ($\times10^{-5}$) absorbing 50% of the cytotoxic activity of 1 μl serum diluted 1:150.

TABLE IV.—*Slopes of absorption curve* of infected cultured cells*

| Infecting viruses | Mean | | | S.d. | |
|---|---|---|---|---|---|
| G and ecotropic G-related MuLV | 1·92 | $\alpha>0\cdot35$ (G vs FMR) | $\alpha>0\cdot10$ (all) | 0·50 | $\alpha>0\cdot10$ (all) |
| FMR-MuLV | 1·80 | $\alpha>0\cdot30$ (FMR vs Xenotropic) | | 0·44 | |
| Xenotropic MuLV | 1·74 | | | 0·33 | |
| All viruses together | 1·82 | | | 0·42 | |

* Mean and variance analysis according to Fisher (1924).

FIG. 3.—Quantitative GCSAa expression on FMR-MuLV-infected murine cells: Number of cells ($\times 10^{-5}$) absorbing 50% of the cytotoxic activity of 1 $\mu$l serum diluted 1:150.

FIG. 4.—Quantitative GCSAa expression on xenotropic-virus-infected xenogenic fibroblasts: Number of cells ($\times 10^{-5}$) absorbing 50% of the cytotoxic activity of 1 $\mu$l serum diluted 1:150.

($NA_{50}$) ranging from $0{\cdot}37 \times 10^5$ cells for SC1-G to $1{\cdot}55 \times 10^5$ cells for 3T3FL(1)G. Similarly, G-related endogenous viruses induced a high GCSAa expression ($NA_{50}$ ranging from $0{\cdot}065 \times 10^5$ cells for SC1-N to $0{\cdot}55 \times 10^5$ cells for 13-3-C).

### In vitro *GCSAa expression induced by MuLV of the FMR group*

Mouse cells infected with M-MuLV expressed an intermediate level of GCSAa activity, the $NA_{50}$ of these cells ranging from $1{\cdot}15 \times 10^5$ cells for SL12P to $3{\cdot}80 \times 10^5$ cells for 3T3-MLV2 (Fig. 3). By contrast, infection with F-MuLV or R-MuLV did not induce GCSAa activity ($NA_{50} \geqslant 5 \times 10^5$ cells for 3T3FL(1)-F, $NA_{50} = 4{\cdot}9 \times 10^5$ cells for BxN-R) with the exception of C3H cells ($NA_{50} = 1{\cdot}58 \times 10^5$ cell for C3H-R). Thus, the GCSAa expression induced by viruses from the FMR-MuLV group was scattered over a wider range than that induced by viruses from the G-MuLV group.

### In vitro *GCSAa expression induced by mouse xenotropic virus*

Xenogenous cell lines producing xenotropic virus from *Mus molossinus* and C57 leaden mouse expressed GCSAa at a level equal to that of G–MuLV-infected mouse cell lines (Fig. 4, $NA_{50} = 1{\cdot}20 \times 10^5$ cells for 8155–MOL and $0{\cdot}95 \times 10^5$ cells for DOG–C57L). Xenotropic virus of NZB induced only an intermediate level of GCSA ($NA_{50} = 1{\cdot}8 \times 10^5$ cells for CL1S2). NIH–Swiss mouse xenotropic virus AT124 is also able to induce GCSAa activity, strongly depending upon the infected cell; from high induction in HUF cells ($NA_{50} = 10^5$ cells) to low induction in DOG cells ($NA_{50} = 4{\cdot}36 \times 10^5$ cells (Fig. 4) and in AT124 cells ($NA_{50} = 3{\cdot}63 \times 10^5$ cells).

### *Relation between GCSAa expression and p30 level*

Since antigenic specificities associated to p30 seem to be involved in GCSAa activity (Ledbetter & Nowinski, 1977; Snyder *et al.*, 1977, Tung *et al.*, 1977), the amount of intracellular p30 was determined in a radioimmunoassay and compared to

FIG. 5.—Relationship between amount of GCSAa and p30 level: (■) G-related MuLV-infected cell line and ——— regression line ($y = 1 \cdot 1055x - 8 \cdot 157$; $r = +0 \cdot 88$; $0 \cdot 01 < 2\alpha < 0 \cdot 05$). (□) FMR-MuLV-infected cell line and - - - regression line ($y = 0 \cdot 0706x - 5 \cdot 6673$; $r = +0 \cdot 10$; $2\alpha > 0 \cdot 10$). (×) Xenotropic-virus-infected cell line and – —— – —— – regression line ($y = 0 \cdot 2624x - 5 \cdot 7714$; $r = +0 \cdot 48$; $2\alpha > 0 \cdot 10$).

the level of GCSAa expressed by the same cells. Statistical analysis of the results is summarized in Fig. 5 ,where $\frac{1}{NA_{50}}$ (*i.e.* the level of GCSAa determinants on the cell surface) was plotted on a log/log scale as a function of p30 level in ng/mg of protein. A significant relationship was found between the level of GCSAa and the level of intracellular p30 when there was infection with virus of the G-MuLV group ($r = +0{\cdot}88$, $0{\cdot}01 < 2\alpha < 0{\cdot}05$). A lower and non-significant relationship was found when cells were infected with mouse xenotropic viruses ($r = +0{\cdot}48$; $2\alpha > 0{\cdot}10$) and no relationship when cells harbouring viruses of the FMR-MuLV group were tested ($r = +0{\cdot}10$; $2\alpha > 0{\cdot}10$).

### DISCUSSION

Despite the presence of several antibody specificities, the anti-(C58NT)D W/Fu rat serum, when appropriately diluted and assayed on mouse E♂G2 target cells, recognized mainly if not exclusively GCSAa, very few Gross-virus-induced lymphoma cells being needed to absorb out the cytotoxic activity, whereas RBL5 cells and 129 thymocytes, which defined the other G-related antigens GCSAb and $G_{IX}$, could not absorb out this cytotoxic activity (Herberman, 1972). Absorption of anti-GCSAa antibodies has however been observed with high numbers of leukaemic cells induced by other agents; this could be due to a nonspecific phenomenon, or to a slight expression of crossreactive moieties on these cells (Herberman, 1972).

When *in vitro* cell lines infected with ecotropic MuLV were assayed for GCSAa expression, it appeared that this antigen can be induced not only by G-type MuLV, but also after infection with FMR-type MuLV or xenotropic viruses, these findings being in agreement with the results reported by O'Donnel & Stockert (1976). Since it has been suggested that at least two subspecificities of the GCSA may exist, one specific for G-MuLV, the other common (O'Donnel & Stockert, 1976), it was then important to determine the fine specificity of the assay used here. Since in contrast with O'Donnel and Stockert's findings (1976) we never observed a partial absorption of our anti-(C58NT)D serum activity by FMR-MuLV or xenotropic-virus-infected cells, it can be assumed that only one GCSAa specificity is being detected in our assay. Moreover, when the percentage of absorption was plotted in log/log scale as a function of the number of absorbing cells (Fig. 1), the straight lines obtained with FMR-MuLV or xenotropic-virus-infected cells showed the same slope than those obtained with G-MuLV-infected cells (Table IV). As hypothesized by Dexter (1976), this was in favour of a homogeneous antigen–antibody system involving the same affinity interactions. Anti-GCSAa antibodies being constant in our system, GCSAa determinant must be homogeneous on the various infected cultured cells. Furthermore, it is not unlikely that the GCSAa specificity we observed with the rat anti-(C58NT)D serum could represent the GCSA subspecificity common to G, FMR and xenotropic MuLV recognized by the C57BL/6 anti-AKR K36 lymphoma, mouse antiserum used in the abovementioned studies (O'Donnel & Stockert, 1976). Thus, although the anti-GCSAa activity of the rat anti-(C58NT)D serum has been described as a G-MuLV typing serum when tested on lymphoma cells, it must be considered only as a MuLV-group-specific antiserum when tested on infected cultured fibroblasts. In addition, the quantitative study of GCSAa expression has clearly shown that a difference in the amount of antigen cannot be used to distinguish *in vitro* infection of cells by a G type or FMR type or a xenotropic virus; a high or intermediate level of GCSAa expression could be induced by G-related MuLV but also by a FMR type or xenotropic MuLV. Furthermore, within these 3 groups of viruses, the level of GCSAa expression varied when a given cell line was infected with different virus isolates or when cell lines were infected by the same virus.

It can then be questioned what molecular species bears the GCSAa activity, and two molecules have been described as precipitated by an anti-(C58NT)D serum from the surface of Gross-virus-induced lymphoma cells: the envelop protein gp70 amd 2 glycosylated precursors of the nucleocapsid (gag) proteins (p30, p15, p12 and p10) with mol. 95,000 and 85,000 ($gp85^{gag}$ and $gp95^{gag}$) (Tung *et al.*, 1977). Although we had no direct evidence, it is not unlikely that, in this experiment, the GCSAa specificity detected by the anti-(C58NT)D serum, though MuLV-group specific, was borne by the $gp95^{gag}$ and $gp85^{gag}$ molecules, since the gp70 is also well expressed on the FMR-MuLV lymphomas cells (Bauer, 1974). Moreover, the GCSA as defined by the mouse antiserum has been described to be borne also by $gp85^{gag}$ and $gp95^{gag}$ (Ledbetter & Nowinski 1977, Snyder *et al.*, 1977, Ledbetter *et al.*, 1978). The discrepancy in specificity of the expression of GCSAa, which appears Gross-virus-specific on MuLV-induced leukaemias but nonspecific Gross virus on MuLV-infected fibroblasts, could be related to a weak or null expression of $gp95^{gag}$ or $gp85^{gag}$ on FMR-MuLV-induced lymphoma cells (Ledbetter *et al.*, 1977), while FMR-virus-infected fibroblasts could express them (Evans *et al.*, 1977; Edwards & Fan 1979; Schultz *et al.*, 1979; Buetti & Diggelman 1980). In accordance with this hypothesis, FMR-MuLV-induced lymphoma cells have been shown not to express p30 antigenic specificities at their surface (Humphrey *et al.*, 1979; Nowinski *et al.*, 1978; Schneider & Hunsmann, 1978).

Antigenic sites associated with p30 molecules being at least partially involved in the GCSAa specificity (Ledbetter & Nowinski, 1977; Snyder *et al.*, 1977), it could be questioned whether the GCSAa expression is related to the level of intracellular nucleocapsid proteins, a reflection of the virus-cell metabolic interaction. A good relationship was found between the GCSAa level and the amount of intracellular p30 when the cells are infected with the G-type viruses, suggesting a homogeneous event. On the contrary no relationship was found during a FMR-MuLV or xenotropic virus infection. The absence of striking evidence for a relationship or independence, when cells were infected by xenotropic virus, would probably be due to the heterogeneity of xenotropic viruses (O'Donnel & Stockert 1976). It is not unlikely that the absence of relationship between the amount of GCSAa and the p30 level in the case of FMR-MuLV infection was due to the MuLV group-specific determination of GCSAa and p30. This overall relationship could be very different from a relationship studied in MuLV-type specific conditions, if, for example, one of these products is the result of two different proviral expressions (one FMR and one endogeneous) as shown recently (Tung & Fleissner 1980). However, this result can be compared with the recent findings that during FMR-MuLV infection the gag products expressed on the cell surface and the intracellular nucleocapsid proteins could result from two different metabolic pathways (Edwards & Fan, 1979; Ledbetter *et al.*, 1978; Schultz *et al.*, 1979) and, obviously, studies with MuLV-type-specific antisera and molecular determination of the antigen are needed.

These *in vitro* findings could explain the different antigenic specificity found between Gross and FMR virus-induced lymphomas, the gag precursor associated GCSA antigens persisting at the cell surface in the first case but not in FMR virus-induced tumours.

We are greatly indebted to Dr J. L. Guenet for providing 129 mice, Dr R. Ravicovich and E. Leftheriotis for providing *in vitro* cell lines, and to F. Pozo and J. P. Portail for their skilful technical assistance. We also thank Dr J. P. Levy for helpful advice and discussions.

### REFERENCES

ARNSTEIN, P., LEVY, J. A., OSHIRO, L. S., PRICE, P. J., SUK, W. & LENNETTE, E. H. (1974) Recovery of murine xenotropic type C virus from C57L mice. *J. Natl. Cancer Inst.*, **53**, 1787.

BASSIN, R. H., TUTTLE, N. & FISCHINGER, P. J. (1970) Isolation of murine sarcoma virus transformed mouse cells which are negative for leukemia virus from agar suspension cultures. *Int. J. Cancer*, **6**, 95.

BAUER, H. (1974) Virion and tumor cell antigens of C type RNA tumor viruses. *Adv. Cancer Res.*, **20**, 275.

BUETTI, E. & DIGGELMANN, H. (1980) Murine leukaemia virus proteins expressed on the surface of infected cells in culture. *J. Virol.*, **33**, 936.

DEXTER, D. L. (1976) A function which describes quantitative absorptions of antibodies by cell surface antigens. *Immunochemistry*, **13**, 801.

EDWARDS, S. A. & FAN, H. (1979) Gag-related polyproteins of Moloney leukemia virus: Evidence for independent synthesis of glycosylated and unglycosylated forms. *J. Virol.*, **30**, 551.

EVANS, L. H., DRESLER, S. & KABAT, D. (1977) Synthesis and glycosylation of polyprotein precursors to the internal core proteins of Friend murine leukemia virus. *J. Virol.*, **24**, 865.

FISHER, R. A. (1924) On a distribution yielding the error function of several wellknown statistics. *Proc. Int. Mathemat. Cong.*, **2**, 805.

FISCHINGER, P. J. & O'CONNOR, T. E. (1970) Productive infection and morphologic alteration of human cells by a modified sarcoma virus. *J. Natl. Cancer Inst.*, **44**, 429.

GEERING, G., OLD, L. J. & BOYSE, E. A. (1966) Antigens of leukemias induced by naturally occurring murine leukemia virus: Their relation to the antigen of Gross virus and other murine leukemia viruses. *J. Exp. Med.*, **124**, 753.

GERLIER, D., GUIBOUT, C. & DORE, J. F. (1977*a*) Highly cytotoxic antisera obtained in W/Fu rats against a syngeneic Gross virus induced lymphoma. *Eur. J. Cancer*, **13**, 855.

GERLIER, D., GUILLEMAIN, B., DORE, J. F. & DUPLAN, J. F. (1977*b*) Expression d'un antigène associé au virus de Gross à la surface de cellules murines productrices d'un oncornavirus des radioleucémies de la souris C57B1/6. *C. R. Acad. Sci.*, (D) (Paris), **284**, 2431.

GISSELBRECHT, S., BASSIN, R. H., GERWIN, B. I. & REIN, A. (1974) Dual susceptibility of a 3T3 mouse cell line to infection by N and B tropic murine leukemia virus: Apparent lack of expression of the Fvl gene. *Int. J. Cancer*, **14**, 106.

GISSELBRECHT, S., POZO, F., DEBRE, P., HUROT, M. A., LACOMBE, M. J. & LEVY, J. P. (1978) Genetic control of sensitivity to Moloney-virus induced leukemias in mice. I. Demonstration of multigenic control. *Int. J. Cancer*, **21**, 626.

GORER, P. A. (1950) Studies in antibody response of mice to tumour inoculation. *Br. J. Cancer*, **4**, 372.

GREENWOOD, F. C., HUNTER, W. M. & CLOVER, J. P. (1963) The preparation of $^{125}$I labelled human growth hormone of high specific radioactivity. *Biochem. J.*, **89**, 114.

HARTLEY, J. W. & ROWE, W. P. (1975) Clonal cell lines from a feral mouse embryo which lack host range restrictions for murine leukemia viruses. *Virology*, **65**, 128.

HARTLEY, J. W., ROWE, W. P. & HUEBNER, R. J. (1970) Host range restriction of murine leukemia viruses in mouse embryo cell cultures. *J. Virol.*, **5**, 221.

HERBERMANN, R. B. (1972) Serological analysis of cell surface antigens of tumors induced by murine leukemia virus. *J. Natl Cancer Inst.*, **48**, 265.

HUMPHREY, D., TSUKAMOTO-ADEY, A., WITTE, O. M., FOX, R., JERABEK, L. & WEISSMAN, I. L. (1979) A serologic comparison of Moloney lymphoma cell surface and Moloney oncornavirus antigens. *J. Immunol.*, **123**, 412.

JAINCHILL, J., AARONSON, S. & TODARO, G. J. (1969) Murine sarcoma and leukemia virus: assay using clonal lines of contact inhibited cells. J. Virol., **4**, 549.

LEDBETTER, J. & NOWINSKI, R. C. (1977) Identification of the Gross cell surface antigen associated with murine leukemia virus infected cells. *J. Virol.*, **23**, 315.

LEDBETTER, J. A., NOWINSKI, R. C. & EISENMAN, R. M. (1978) Biosynthesis and metabolism of viral proteins expressed on the surface of murine leukemia virus-infected cells. *Virology*, **91**, 116.

LEDBETTER, J., NOWINSKI, R. C. & EMERY, S. (1977) Viral proteins expressed on the surface of murine leukemia cells. *J. Virol.*, **22**, 65.

LIEBER, M., SHERR, C., POTTER, M. & TODARO, G. (1975) Isolation of C type viruses from the asian feral mouse *Mus musculus molossinus*. *Int. J. Cancer*, **15**, 211.

LEVY, J. A. (1973) Xenotropic viruses: Murine leukemia viruses associated with NIH SWISS, NZB and other mouse strains. *Science*, **182**, 1151.

LEVY, J. A. & PINCUS, T. (1970) Demonstration of biological activity of a murine leukemia virus of New Zealand Black mice. *Science*, **170**, 326.

LEVY, J. P., LECLERC, J. C., VARET, B. & OPPENHEIM, E. (1968) Study of the antigenic specificity of Graffi leukemic cells. *J. Natl Cancer Inst.*, **41**, 743.

MAMOUN, R., GUILLEMAIN, P., ASTIER, T., PORTAIL, J. P., LEGRAND, E. & DUPLAN, J. F. (1978) Production and analysis of a viral complex derived from a radiation induced leukemia virus of C57BL mouse. *Int. J. Cancer*, **22**, 98.

MCCOY, J. L., FEFER, A. & GLYNN, J. P. (1967) Comparative studies on the nduction of transplantation resistance in BALB/c and C57BL/6 mice in three murine leukemia systems. *Cancer Res.*, **27**, 1743.

NOWINSKI, R. C., EMERY, S. & LEDBETTER, J. (1978) Identification of an FMR cell surface antigen associated with murine leukemia virus infected cells. *J. Virol.*, **26**, 805.

O'DONNEL, P. V. & STOCKERT, E. (1976) Induction of $G_{IX}$ antigen and Gross cell surface antigen aftrr infection by ecotropic and xenotropic murine leukemias virus *in vitro*. *J. Virol.*, **20**, 545.

OLD, L. J., BOYSE, E. A. & STOCKERT, E. (1965) The G(Gross) leukemia antigen. *Cancer Res.*, **25**, 813.

REZNIKOFF, C. A., BRANKOW, D. W. & HEIDELBERGER, C. (1973) Establishment and characterization of a cloned line of C3H mouse embryo cells sensitive to postconfluence inhibition of division. *Cancer Res.*, **33**, 3231.

SCHAFER, W. & SEIFERT, E. (1968) Production of a potent complement fixing murine leukemia virus antiserum from the rabbit and its reactions with various types of tissue culture cells. *Virology*, **35**, 323.

SCHNEIDER, J. & HUNSMANN, G. (1978) Surface expression of murine leukemia virus structural polypeptides on host cells and the virion. *Int. J. Cancer*, **22**, 204.

SCHULTZ, A. M., RABIN, E. H. & OROSZLAN, S. (1979) Post translational modification of Rauscher leukemia virus precursor polyproteins encoded by the gag gene. *J. Virol.*, **30**, 255.

SNYDER, H. W., STOCKERT, E. & FLEISSNER, E. (1977) Characterization of molecular species carrying Gross cell surface antigen. *J. Virol.*, **23**, 302.

STEPHENSON, J. R. & AARONSON, S. A. (1972) A genetic locus for inducibility of C-type virus in BALB/c cells: The effect of a non linked regulatory gene on detection of virus after chemical activation. *Proc. Natl Acad. Sci.*, **69**, 2798.

TODARO, G. J., ARNSTEIN, P., PARKS, W. P., LENNETTE, E. H. & HUEBNER, R. J. (1973) A type C virus in human rhabdomyosarcoma cells after inoculation into NIH Swiss mice treated with antithymocyte serum. *Proc. Natl Acad. Sci.*, **70**, 859.

TODARO, G. J. & GREEN, H. (1963) Quantitative studies of the growth of mouse embryo cells in culture and their development into established lines. *J. Cell Biol.*, **17**, 299.

TUNG, J. S. & FLEISSNER, E. (1980) Amplified env and gag products on AKR cells. Origin from different murine leukemia virus genomes. *J. Exp. Med.*, **151**, 975.

TUNG, J. S., PINTER, A. & FLEISSNER, E. (1977) Two species of type C viral core polyprotein on AKR mouse leukemia cells. *J. Virol.*, **23**, 430.

WRIGHT, B. J., O'BRIEN, P. A., SHIBLEY, G. P., MAYYASI, S. A. & LASFARGUES, J. C. (1967) Infection of an established mouse bone marrow cell line (JLS.V.9) with Rauscher and Moloney murine leukemia viruses. *Cancer Res.*, **27**, 1672.